# Atrial Strain Analysis Predicts Atrial Arrhythmia Recurrence Following Cavotricuspid Isthmus Ablation of Typical Atrial Flutter

**DOI:** 10.3390/jcm14155247

**Published:** 2025-07-24

**Authors:** Giulia Iannaccone, Roberto Scacciavillani, Francesca Graziani, Filippo Tusa, Carlo Piccinni, Francesca Augusta Gabrielli, Maria Lucia Narducci, Francesco Perna, Massimiliano Camilli, Maria Chiara Meucci, Rocco A. Montone, Gianluigi Bencardino, Gaetano Antonio Lanza, Gemma Pelargonio, Antonella Lombardo

**Affiliations:** 1 Department of Cardiovascular Sciences-CUORE, Catholic University of the Sacred Heart, 00168 Rome, Italy; giuliaiannaccone1992@gmail.com (G.I.); roberto.scacciavillani@gmail.com (R.S.); filippo.tusa@icatt.it (F.T.); carlo.piccinni@icatt.it (C.P.); marialucia.narducci@policlinicogemelli.it (M.L.N.); massimiliano.camilli1@policlinicogemelli.it (M.C.); roccoantonio.montone@policlinicogemelli.it (R.A.M.); gaetanoantonio.lanza@policlinicogemelli.it (G.A.L.); gemma.pelargonio@policlinicogemelli.it (G.P.); antonella.lombardo@policlinicogemelli.it (A.L.); 2 Department of Cardiovascular Sciences-CUORE, Fondazione Policlinico Universitario A. Gemelli IRCCS, 00168 Rome, Italy; francescaaugusta.gabrielli@policlinicogemelli.it (F.A.G.); francesco.perna@policlinicogemelli.it (F.P.); mariachiara.meucci@guest.policlinicogemelli.it (M.C.M.); gianluigi.bencardino@policlinicogemelli.it (G.B.)

**Keywords:** atrial flutter, arrhythmia recurrence, strain echocardiography, speckle tracking analysis, cavotricuspid isthmus ablation

## Abstract

**Background:** This study aimed to evaluate the effectiveness of right and left atrial strain reservoir (RASr and LASr) in predicting the recurrence of atrial arrhythmias (AAs) following cavotricuspid isthmus ablation (CTIA) for typical atrial flutter (AFL). **Methods:** We retrospectively enrolled consecutive patients with AFL who had undergone CTIA. Transthoracic echocardiography was conducted within one month before the procedure, and atrial two-dimensional speckle tracking analysis was performed offline. **Results:** Sixty-two subjects were evaluated (mean age 64.8 ± 13.2 years, 29% females). At a median follow-up of 12.1 months, AA recurrence occurred in 21 subjects (33.8%). The study endpoint occurred mainly among females (*p* = 0.021) and patients with lower RASr and LASr values (both *p* < 0.001). In Cox regression analysis, RASr and LASr remained independent predictors of AA recurrence (*p* = 0.02 and *p* = 0.03, respectively). In ROC curve analysis, RASr and LASr showed a similar and satisfactory ability to predict AA recurrence with optimal cut-off values of 16.8% and 17.7%, respectively. In survival analysis, RASr > 16.8% and LASr > 17.7% were associated with significantly higher freedom from AAs during follow-up (log rank *p* = 0.001 and *p* = 0.002, respectively). **Conclusions:** The results of this study suggest that pre-CTIA atrial speckle tracking analysis may aid in identifying AFL patients at an increased risk of AA recurrence, allowing for more frequent follow-up visits and extended antiarrhythmic therapy.

## 1. Introduction

Typical cavotricuspid isthmus-dependent atrial flutter (AFL) is a common arrhythmia that may affect patients’ quality of life and carries a risk of thromboembolic events similar to that of atrial fibrillation (AF) [1]. The electrophysiological substrate underlying AFL is well defined, and since rhythm control is rarely achieved with medical therapy, cavotricuspid isthmus ablation (CTIA) is a widely recognized first-line therapy for AFL, demonstrating an acute success rate exceeding 90% for initial ablation and a minimal recurrence rate [2]. The assessment of left and right atrial function using strain echocardiography has recently emerged as a valuable tool for predicting AF recurrence following electrical cardioversion or transcatheter ablation [3,4]. However, as far as we know, no similar investigations have been conducted in individuals with AFL. Identifying potential predictors of atrial arrhythmia (AA) recurrence after catheter ablation for AFL could be clinically significant in determining patients who may benefit from closer monitoring, continued antiarrhythmic therapy, and repeat procedures. The present study aimed to assess the ability of left and right atrial strain analysis to predict AA recurrence after CTIA in typical AFL.

## 2. Materials and Methods

### 2.1. Study Design and Population

We retrospectively screened all patients referred for typical AFL without a previous known history of AF who underwent CTIA for the first time at our center (Fondazione Policlinico Universitario Agostino Gemelli IRCCS, Rome) from June 2020 to May 2023 (n = 132). We excluded patients (1) with a follow-up period shorter than 3 months (n = 27), (2) with no available echocardiographic images (n = 30), and (3) with acoustic windows inadequate for strain analysis (n = 13) (Figure S1). After the index ablation procedure, AA recurrence was established, with in-office visits scheduled at 3, 6, and 12 months and every 6 months thereafter. At every appointment, a detailed medical history was gathered, a physical check-up was performed, and a 12-lead ECG and a 24 h Holter monitor recording were carried out. Hospital admission, either for arrhythmic disorders or due to other causes, was also evaluated. AA recurrence was defined as the documentation of AFL or atrial fibrillation (AF) lasting more than 30 s during planned follow-up visits or hospitalizations. The study was approved by the Institutional Ethical Committee (reference ABT-CIP-10316, approval date 29 June 2020) and conforms to the ethical guidelines of the 1975 Declaration of Helsinki.

### 2.2. Echocardiography

All patients underwent standard two-dimensional echocardiography within one month before CTIA. Ultrasound images were obtained with patients resting in the left lateral position, using a commercially available ultrasound system (Philips Epiq 7: Philips, Amsterdam, The Netherlands) equipped with an M5S probe. Two-dimensional, color, pulsed-wave, and continuous-wave Doppler data were obtained in parasternal, apical, subcostal, and suprasternal windows. As suggested by current guidelines [5], at least 3 cardiac cycles were acquired in the case of sinus rhythm and 5 in the case of AFL rhythm during the echocardiographic evaluation. The left ventricular ejection fraction (LVEF) was calculated using the modified Simpson method in apical 4- and 2-chamber views (A4Ch and A2Ch). The right ventricle (RV) mid-diameter was calculated in diastole, and right ventricular function parameters were assessed, including the tricuspid annulus plane systolic excursion (TAPSE) and right ventricular fractional area change (RVFAC). The pulmonary arterial systolic pressure (PASP) was calculated as the sum of the tricuspid regurgitation peak velocity on continuous-wave Doppler and the estimated right atrial pressure. The left atrial volume index (LAVi) was calculated by measuring the maximum left atrium (LA) volume before mitral valve opening in A4Ch and A2Ch according to the biplane disk summation technique and indexed for the body surface area [5]. Similarly, the right atrial volume index (RAVi) was assessed by measuring the maximum right atrium (RA) volume before tricuspid valve opening and indexed for the body surface area. [5]. LA and RAstrain analysis was conducted offline by an experienced operator using a vendor-independent dedicated software (TomTec Arena 2020 Imaging Systems, Unterschleissheim, Germany,). The endocardial border of LA and RA were automatically identified over one frame and automatically tracked throughout the cardiac cycle [6,7]. The adequacy of tracking was verified manually, and the region of interest was adjusted to achieve optimal tracking, including the entire myocardial wall. LA strain analysis was performed, averaging the values obtained from A4Ch and A2Ch, while RA strain values were assessed from A4Ch according to the latest consensus [6]. Strain measurements reflected the peak% change in the length of the LA myocardium during the cardiac cycle. Considering the high prevalence of patients in AFL during the echocardiographic evaluation (n = 21, 41%), only the RA reservoir strain (RASr) and LA reservoir strain (LASr) were assessed. The inter- and intra-observer variability of echocardiographic measurements among the operators of our Echocardiographic Laboratory has been reported previously [8].

### 2.3. Cavotricuspid Isthmus Ablation Procedure

Local anesthesia and conscious sedation were performed before all the ablation procedures. The endocardial high-density electroanatomical voltage mapping of the right atrium and especially of the cavotricuspid isthmus (CTI) was performed using a multi-electrode mapping catheter (Intella NAV Orion, Boston Scientific, Cambridge, MA, USA; PentaRay, Biosense Webster, Inc., Diamond Bar, CA, USA; HD Grid, Abbott, Abbott, North Chicago, IL, USA) with one of three electroanatomical mapping systems, depending on the operator’s preference: Rhythmia (Boston Scientific, Cambridge, MA, USA), CARTO 3 (Biosense Webster, Inc., Diamond Bar, CA, USA), or EnSite NavX (Abbott, North Chicago, IL, USA). The radiofrequency ablation of the CTI with an open irrigated catheter was performed in all cases: Intella NAV MiFi OI (Boston Scientific, Cambridge, MA, USA); Thermocool SmartTouch SF (Biosense Webster, Inc., Diamond Bar, CA, USA), or Flexability (Abbott, North Chicago, IL, USA). Acute procedural success was defined by the termination of the clinical arrhythmia and bidirectional block across the ablation line proven by pacing maneuvers. Finally, atrial fibrillation inducibility was tested in all patients, as suggested by recent evidence [9] following a protocol of atrial bursts until refractoriness or up to a cycle length of 200 ms. Pulmonary vein isolation was therefore performed if sustained atrial fibrillation was induced (>30 s or requiring electrical or pharmacological cardioversion for termination).

### 2.4. Statistical Analysis

Continuous variables were expressed as the mean ± standard deviation (SD) or as the median (interquartile range, IQR), according to data distribution, assessed by the Kolmogorov–Smirnov test. Categorical data were expressed as a number (percentage). Continuous variables were compared using an unpaired Student’s *t*-test or Mann–Whitney U test, and categorical data were evaluated using the Chi2 test or Fisher’s exact test, as appropriate. A *p*-value < 0.05 was considered statistically significant. Cox regression multivariate analysis for AA recurrence was performed, including all significant variables at univariate analysis and without significant multicollinearity, assessed by the variance inflation index. Receiver-operating characteristic (ROC) curve analysis was used to estimate the overall predictive accuracy of atrial strain values to predict AA recurrence by evaluating the area under the curve (AUC) and the respective 95% confidence interval (CI). ROC curves were also used to establish the optimal cut-off value for both RASr and LASr to predict AA recurrence. A post hoc power analysis based on ROC curves was performed to assess the sample size adequateness. The cumulative event-free survival rates for the study population, stratified by the presence of atrial strain values higher or lower than the cut-off values, were calculated using the Kaplan–Meier method. As secondary analyses, differences between patients in AFL and in sinus rhythm during the echocardiographic evaluation, between those who received isolated CTIA and those who underwent concomitant AF ablation, and between those with AA recurrence as AFL or AF were assessed, as appropriate. SPSS (SPSS version 26, Inc., Chicago, IL, USA) statistical software was used to perform all the analyses.

## 3. Results

Sixty-two patients were finally enrolled [mean age, 64.8 ± 13.2 years; 18 females (29%)], among whom 58.4% had hypertension, 14.5% had diabetes, 9.7% had coronary artery disease, 14.5% were active smokers, and 9.7% had chronic obstructive pulmonary disease (Table 1). In a total of 27 patients (43.5%), AF was induced during the procedure, and the concomitant ablation of both AFL and AF was performed. The clinical and echocardiographic baseline features of the population are depicted in Table 1. LV and RV function were roughly normal in most patients (LVEF 50.5% ± 13.1, TAPSE 18.2 ± 4.1 mm). Similarly, LA and RA were mildly enlarged in most patients [LAVi 40.1 ± 15.9 mL/m^2^, RAVi 30.2 mL/m^2^ (21.4–41.5)]. The mean LASr and RASr values were 18.8% ± 8.7 and 17.6% ± 6.9, respectively. At least moderate mitral and tricuspid regurgitation were detected in 7 and in 6 patients, respectively. Among these, one patient presented moderate-to-severe mitral and tricuspid regurgitation, and another presented severe tricuspid regurgitation, while the degree of atrioventricular valve regurgitation was moderate in the others. After a median follow-up time of 12 months (IQR: 7–18), AAs occurred in 21 subjects (33.8%), of which 11 (52%) experienced typical AFL recurrence and 10 (48%) experienced AF.

### 3.1. Predictors of Atrial Arrhythmia Recurrence

**Figure 1:**
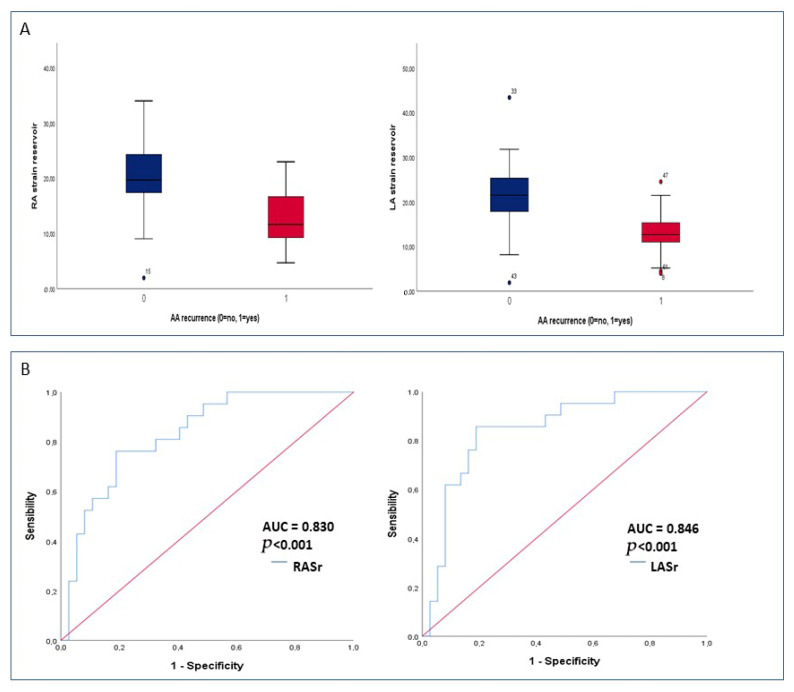
Predictors of AA recurrence. (**A**) Box plot with median and interquartile ranges of RA strain reservoir and LA reservoir in patients with AA recurrence vs. those without. Blue bar = no AA recurrence; red bar = AA recurrence (**B**) ROC curve analysis was performed to compare the ability to predict atrial arrhythmia recurrence of RASr and LASr values. Abbreviations: AUC, area under the curve; LA, left atrial; LASr, left atrial strain reservoir; RA, right atrial; RASr, right atrial strain reservoir; ROC, receiving operating characteristic.

**Figure 2:**
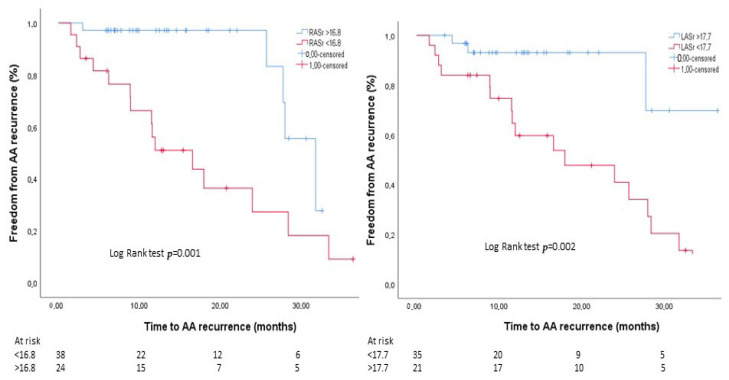
Kaplan–Meier curves based on RASr and LASr cut-off values individuated by ROC curve analysis (16.8% and 17.7%, respectively). Abbreviations: AAs, atrial arrhythmias; LASr, left atrial strain reservoir; RASr, right atrial strain reservoir.

**Figure 3:**
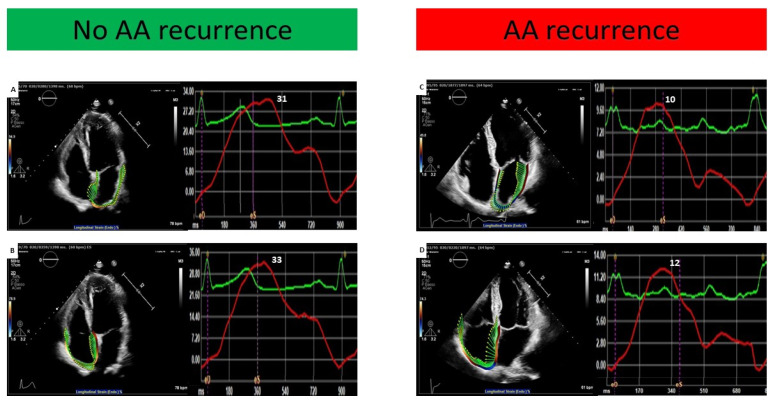
Echocardiographic images with atrial strain analysis performed in two patients included in our study. On the left, the pre-AFL ablation atrial strain analysis of a patient who did not experience AAs at follow-up shows normal values of LASr (**A**) and RASr (**B**). On the right, pre-AFL ablation atrial strain analysis of a patient with documented AA recurrence at follow-up shows low LASr (**C**) and RASr (**D**) values. Abbreviations: AAs, atrial arrhythmias; AFL, atrial flutter; LASr, left atrial strain reservoir; RASr, right atrial strain.

There were no significant differences as regards most of the baseline clinical features between patients with and without AA recurrence, with the exception of a higher occurrence among females (*p* = 0.021) and a borderline significant higher prevalence among those who underwent concomitant AF ablation (*p* = 0.051). In addition, the two populations did not differ in standard 2D echocardiographic parameters of chamber dimensions and ventricular function (Table 1). Of note, RASr and LASr were found to be significantly lower in patients who experienced AA recurrence (12.8 ± 5.3 vs. 20.3 ± 6.3 and 13.5 ± 5.2 vs. 22.1 ± 8.5, respectively, both *p* < 0.001) (Table 1, Figure 1A). Univariate Cox regression analysis showed that only RASr and LASr were predictive of the study endpoint (Table 2). In multivariate Cox regression analysis, both RASr and LASr were confirmed to be independent predictors of AA recurrence (HR 0.904, 95% CI [0.831–0.984], *p* = 0.02 and HR 0.919, 95% CI [0.852–0.992], *p* = 0.03, respectively) (Table 2). In ROC curve analysis, RASr and LASr showed a similar and satisfactory ability to predict AA recurrence [AUC 0.830 (95% CI 0.725–0.935, *p* < 0.001) and AUC 0.846 (95% CI 0.741–0.950, *p* < 0.001), respectively] (Figure 1B). A cut-off value of 16.8% for RASr and of 17.7% for LASr yielded the highest sensitivity and specificity for the prediction of AA recurrence (76% and 88% for RASr and 86% and 88% for LASr, respectively). A post hoc power analysis based on ROC curves showed a statistical power of 97.9% for RASr and 98.8% for LASr, confirming that the study was adequately powered to detect the observed discriminative performance of these parameters. The baseline clinical and echocardiographic characteristics of the overall study population stratified by RASr and LASr cut-off values are reported in Tables S1 and S2. The only statistically significant differences were a higher prevalence of diabetes among patients with RASr < 16.8% (*p* = 0.013) and a larger mean LAVi in the subgroup with LASr < 17.7% (*p* = 0.036). As shown in Figure 2, patients with RASr and LASr above the cut-off values presented significantly higher event-free survival rates in comparison with their counterparties (log-rank test *p* = 0.001 and 0.002, respectively). Figure 3 illustrates the bi-atrial strain analysis performed in two patients included in our study, one who experienced AA recurrence at follow-up and one who did not.

### 3.2. Differences Between Patients in AFL and in Sinus Rhythm During Echocardiography

Table 3 illustrates the differences between patients in AFL and in sinus rhythm during echocardiographic evaluation. Patients in AFL presented a higher LAVi (*p* < 0.001), higher RAVi (*p* = 0.015), and lower LVEF (*p* = 0.005). In contrast, there were no significant differences as regards LASr (*p* = 0.189) and RASr (*p* = 0.159). Furthermore, there was not statistically significant difference in terms of AFL recurrence at follow-up among the two groups (*p* = 0.516).

### 3.3. Comparison Between Patients Who Received Concomitant AF Ablation and Those Who Did Not

Table 4 shows a comparison between patients who received isolated CTIA and those who underwent concomitant AF ablation. The two groups did not present statistically significant differences in terms of sex, age, hypertension, or history of CAD and COPD. Of note, patients who received also AF ablation were more likely to be discharged on AADs (*p* = 0.013). No significant differences were found as regards standard two-dimensional echocardiographic parameters, nor in terms of RASr (17.2% ± 6.7 vs. 18% ± 7.3, *p* = 0.790) and LASr (18.9% ± 6.7 vs. 18.5% ± 10.7, *p* = 0.444).

### 3.4. Comparison Between Patients with AFL and AF Recurrence

Table 5 shows the comparison between patients who experienced AFL and AF recurrence. The two groups did not present statistically significant differences in terms of baseline clinical characteristics. Interestingly, patients who developed AF at follow-up presented more frequently with diabetes compared to their counterparts (*p* = 0.02). Of note, patients with AF at follow-up were not more likely to have undergone concomitant AF ablation during CTIA (*p* = 0.864). In addition, no significant differences were evidenced as regards standard echocardiographic parameters of ventricular function and chamber volumes, including the dimensions of the right (*p* = 0.654) and the left atrium (*p* = 0.512), nor in terms of RASr (12.5% ± 4.8 vs. 13.2% ± 6, *p* = 0.863) and LASr (LASr 13.2% ± 5.9 vs. 12.6 ± 4.5, *p* = 0.973).

## 4. Discussion

To the best of our knowledge, our study is the first one to investigate the ability of bi-atrial strain to predict AA recurrence following CTIA in typical AFL. The main findings of our analysis are as follows: (1) both RASr and LASr are independent predictors of AA recurrence in patients with typical AFL undergoing CTIA and (2) pre-CTIA RASr and LASr are similarly impaired in patients who develop AAs at follow-up.

Several echocardiographic and cardiac magnetic resonance studies have investigated the utility of atrial strain analysis in different clinical settings. In particular, LA function assessment has been demonstrated to hold important prognostic significance in a great variety of cardiac disorders [8,10,11] and to improve the non-invasive estimation of LV filling pressure [12,13]. RA strain analysis, on the other hand, has mainly been studied in pulmonary hypertension [14,15] but has recently been revealed to also represent a useful tool in predicting outcomes in various phenotypes of heart failure [16,17] and the demand for right ventricular assistance in left ventricular assist device recipients [18].

Over the last several years, atrial strain analysis has also been investigated in the setting of atrial arrhythmias, with most available data focused on AF. In fact, there is plenty of evidence that LA strain is impaired in AF and may help to identify subjects at increased recurrence risk. Brás et al. [19] demonstrated that in 78 patients with either paroxysmal, persistent, or long-standing persistent AF, reduced LASr was an independent predictor of arrhythmic recurrence following catheter ablation. Similar results have also been obtained in AF patients who have undergone electrical cardioversion [3].

In all the studies, the ability of LA strain values to predict AF recurrence was independent of the LA dimensions. These results further underline the promising role of strain analysis in the early stages of cardiac diseases, as it is well known that functional abnormalities usually precede volumetric changes in the cardiac chambers. In addition, patients with paroxysmal AF often present normal atrial volumes.

Concerning AFL, two CMR studies [20,21] showed that in patients undergoing AF ablation, reduced pre-procedural LASr and RASr values were associated, respectively, with concomitant left AFL [20] and typical AFL [21]. In addition, Jou et al. [22] demonstrated a significant post-procedural improvement in RA volume index and LASr in patients undergoing CTI-dependent AFL ablation.

In our study, LASr and RASr were identified as independent predictors of AA recurrence following CTIA for typical AFL, with cut-off values of 17.7% and 16.8%, respectively, which are consistent with previous reports (LASr was 18% in a study by Brás et al. [19], and RASr was 15% in a study by Tomaselli et al. [23]).

The finding of bi-atrial dysfunction in our population, even if primarily affected by an arrhythmia emerging from the RA, is not surprising. In fact, over recent years, the importance of both atria dysfunction in the occurrence and recurrence of different types of AAs has been well documented. Indeed, in a study by Tomaselli et al. [23], RASr was found to be a stronger predictor of AF recurrence following electrical cardioversion than LASr. Furthermore, Egbe et al. [24] demonstrated that in patients with repaired Tetralogy of Fallot, lower RASr values are associated with new-onset AAs. Interestingly, the aforementioned study by Jou et al. [22] highlighted an improvement in LASr in typical AFL patients after the restoration of sinus rhythm through catheter ablation. Of note, it is not uncommon for subjects who underwent ablation for AFL to develop AF at follow-up [25], and vice versa [26].

These results are consistent with the common consideration of AF and right AFL as “fellows-travellers” [27,28], which share several features [29,30] in terms of pathology, dynamic electrophysiological interactions, and clinical context, even if the electrophysiologic/anatomic substrates lay in different atria. In fact, AFL often develops following episodes of AF, as the chaotic electrical activity during AF can create a functional block line between the superior and inferior vena cava, an essential condition for the stable re-entrant circuit that causes AFL [31]. Conversely, fast AFL with very short cycle lengths can lead to fibrillatory conduction, resulting in AF [32,33]. Thus, AF can promote AFL and vice versa, depending on the formation of conduction blocks and the speed of re-entrant circuits [30,31,32,33]. This shared pathophysiology explains why the two often coexist in patients and can transition into one another.

Therefore, it is not surprising that in our population, AF was induced in 43.5% of patients and that AF accounted for a significant number of AA recurrence cases, similarly to results reported in other studies [9,25,34,35,36]. In particular, a study by Hsieh et al. showed AF inducibility in 42% of patients without a previous history of AF undergoing CTIA for AFL [25], with AF occurrence rates during follow-up around 20% [25]. Indeed, in the FLUTIFIB study enrolling 100 AFL patients with no AF history undergoing CTIA, the authors found AF episodes in 77% of patients via implantable loop recorder monitoring after a median follow-up of 24 months [36]. Moreover, although we selected a population without a known history of AF, we cannot state with absolute certainty that none of these patients had experienced undocumented episodes of AF in the past. Even though the inclusion of these patients may have produced a partially mixed population, we decided not to exclude them from the main analysis, in order to both reduce the selection bias and extend the applicability of our results to a wider population. Notably, in our population, there were no significant clinical or echocardiographic differences between patients with AFL and those with AF at follow-up, with the exception of a higher incidence of AF among diabetic individuals, and concomitant AF ablation during CTIA was not revealed to be an independent predictor of arrhythmic recurrence.

Another finding worthy of discussion is the relatively high AFL recurrence rate that we observed in our patients (11, 17.7%); this rate is reported by current guidelines to be around 10% [37] and usually does not exceed 13–14% [38,39]. This discrepancy is likely primarily due to selection bias, as more than half of the original study population had to be excluded due to not meeting the inclusion criteria. Indeed, we did not enroll 27 patients due to a lack of follow-up data, and in our experience, patients without current medical issues (i.e., arrhythmic recurrence) are more prone to skip visits. Additional factors, such as procedural variability and inter-operator differences, must be considered. Finally, a referral bias may have also partially contributed, with our hospital being a tertiary care center with a high prevalence of multimorbid patients. In fact, higher AFL recurrence following CTIA has been reported among more complex populations (up to 38%) [40,41,42]. Therefore, we can conclude that the available evidence shows a wide variability among studies in terms of arrhythmic recurrence following AFL ablation, probably in part depending on the specific characteristics of the patient population and on the type of rhythm monitoring adopted.

To conclude, on the basis of the results of our study, atrial strain analysis before CTIA in typical AFL may help individuate those patients at increased risk of AAs during follow-up. Therefore, as a possible clinical application of our findings, this simple echocardiographic analysis could be used as an additional tool to establish the modality and timing of follow-up and to determine the indications for prolonged antiarrhythmic therapy. Moreover, it may be useful to provide the operator performing the ablation with a non-invasive pre-procedural assessment of the potential presence of a highly arrhythmogenic substrate related to atrial fibrosis. Of note, our study needs to be considered hypothesis-generating, as the small sample size limits the generalizability of our findings and further, larger studies are needed to confirm the potential role of pre-CTIA in AFL atrial strain analysis in predicting AA recurrence.

### Limitations

We recognize that the current study has certain limitations. Firstly, the retrospective design and the limited patient population from a single center may have impacted the results of our analysis. As mentioned above, referral bias needs to be taken into account. Moreover, selection bias cannot be excluded, considering that a large percentage of the initial study population was not enrolled. However, we decided to apply strict exclusion criteria in order to avoid suboptimal strain analysis and a short follow-up period. In addition, the high number of patients in AFL during the echocardiographic evaluation limited a more thorough analysis of the different atrial function phases. However, we did not exclude this subgroup, in order to improve the applicability of our results to daily clinical practice, as it is not unusual to perform pre-CTIA echocardiography while the patient is in AFL. Moreover, prior studies have shown that although strain values, particularly reservoir strain, may be lower during atrial fibrillation or flutter compared to sinus rhythm [23], these differences are often modest and may not reach statistical significance, especially when multiple cardiac cycles are averaged to account for beat-to-beat variability [23,43,44]. Moreover, atrial strain measured during arrhythmia remains feasible and clinically meaningful, and its prognostic value has been preserved across rhythms in various populations [23,43,44]. Importantly, in our cohort, there were no statistically significant differences in terms of atrial strain values or recurrence in subjects in sinus rhythm vs. AFL during echocardiography, suggesting comparable atrial functional assessment across rhythms. Furthermore, another relevant limitation of our study is that post-procedural atrial strain analysis was not performed, as a complete echocardiographic examination was not registered after CTIA in most cases. However, as atrial ablations induce various degrees of reversible “atrial stunning” during the first days following the procedure, it is likely that the reliability of atrial strain values obtained in this period is low. Similarly, we could not retrieve longer follow-up echocardiographic images in most cases. This prevented us from exploring the occurrence of adverse atrial remodeling, which may have contributed to AA recurrence in some cases. Likewise, we could not precisely assess other potential mechanisms behind AA recurrence (i.e., incomplete CTI block for AFL recurrence or pulmonary vein reconnection and the presence of non-PV triggers for AF recurrence), since we did not perform a redo procedure on any of our patients. Finally, event adjudication relied predominantly on scheduled follow-up visits and unplanned hospital admissions to identify AA recurrences, rather than employing continuous rhythm monitoring techniques such as implantable loop recorders or other implantable cardiac devices. This methodological approach may have led to an underestimation of the true incidence of asymptomatic or short-lived episodes of AA recurrence, nevertheless favoring clinically relevant episodes.

## 5. Conclusions

In our cohort of patients with typical AFL, lower pre-CTIA values for both RASr and LASr strain were identified as independent predictors of AA recurrence after a median follow-up of 12 months. Thus, preoperative atrial strain may assist in identifying patients at a greater risk of AA recurrence, who could benefit from more frequent follow-up visits and extended antiarrhythmic therapy. Larger studies are needed to validate our findings.

## Figures and Tables

**Table 1:** Baseline population characteristics and comparison between subgroups with and without atrial arrhythmia recurrence.

	Overall Population (n = 62)	No AA Recurrence (n = 41)	AA Recurrence (n = 21)	*p*-Value (Sig.2-Tailed)
*Clinical characteristics*				
Female sex (n, %)	18 (29%)	8 (19.5%)	10 (47.6%)	0.021
Age (years)	64.8 ± 13.2	63.4 ± 13.9	67.4 ± 11.4	0.356
BMI (kg/m^2^)	26.9 ± 4.5	26.7 ± 4.4	27.5 ± 4.7	0.484
Hypertension (n, %)	34 (58.4%)	23 (56%)	11 (52.4%)	0.781
Diabetes mellitus (n, %)	9 (14.5%)	5 (12.2%)	4 (19%)	0.469
Current smoker (n, %)	9 (14.5%)	5 (12.2%)	4 (19%)	0.469
CAD (n, %)	6 (9.7%)	6 (14.6%)	0 (0%)	0.065
COPD (n, %)	6 (9.7%)	4 (9.8%)	2 (9.5%)	0.977
Concomitant AF ablation (n, %)	27 (43.5%)	14 (34.1%)	13 (61.9%)	0.051
Beta-blockers (n, %)	49 (79%)	32 (78%)	17 (80.9%)	0.929
AADs (n, %)	22 (35.5%)	11 (26.8%)	11 (52.4%)	0.055
Creatinine (mg/dl)	1.1 ± 0.4	1.1 ± 0.3	1.2 ± 0.5	0.944
*ECG features*				
HR (bpm)	93.5 ± 33.2	91.9 ± 31.7	96.5 ± 36.7	0.704
QRS (ms)	119.2 ± 31	119.5 ± 34.4	118.6 ± 23.8	0.577
*Echocardiography*				
LVEDV (mL)	99.2 ± 31.1	101 ± 33.8	95.7 ± 25.1	0.679
LVEF (%)	50.5 ± 13.1	50.8 ± 13.9	49.7 ± 11.7	0.579
LAVi (mL/m^2^)	40.1 ± 15.9	39.6 ± 13.4	41.1 ± 14.4	0.559
LASr (%)	18.8 ± 8.7	22.1 ± 8.5	13.5 ± 5.2	<0.001
TAPSE (mm)	18.2 ± 4.1	18.6 ± 3.9	17.6 ± 4.4	0.295
PASP (mmHg)	33.2 ± 8.5	32.3 ± 9	32.2 ± 4.4	0.914
RVFAC (%)	32.3 ± 11.1	30.8 ± 9.8	34.9 ± 13.1	0.203
RV mid-diameter (mm)	29.8 ± 6.2	30.2 ± 7	29.1 ± 4.4	0.949
RAVi (mL/m^2^)	30.2 (21.4–41.5)	31.3 (21.6–40.3)	35.6 (20.8–50.5)	0.489
RASr (%)	17.6 ± 6.9	20.3 ± 6.3	12.8 ± 5.3	<0.001
At least moderate MR (n, %)	7 (11.3%)	3 (7.3%)	4 (19%)	0.167
At least moderate TR (n, %)	6 (9.7%)	2 (4.9%)	4 (19%)	0.074

Abbreviations: AA, atrial arrhythmia; AADs, antiarrhythmic drugs; AF, atrial fibrillation; BMI, body mass index; CAD, coronary artery disease; COPD, chronic obstructive pulmonary disease; HR, heart rate; LASr, left atrial strain reservoir; LAVi, left atrial volume indexed; LVEDV, left ventricular end diastolic volume; LVEF, left ventricular ejection fraction; MR, mitral regurgitation; PASP, pulmonary artery systolic pressure; RAVi, right atrial volume indexed; RASr, right atrial strain reservoir; RV, right ventricle; RVFAC, right ventricle fractional area; TAPSE, tricuspid annulus planar systolic excursion; TR, tricuspid regurgitation.

**Table 2:** Univariate and multivariate analysis to predict atrial arrhythmia recurrence.

	Univariable		Multivariable	
	Hazard Ratio (95% CI)	*p*-Value	Hazard Ratio (95% CI)	*p*-Value
*Clinical variables*				
Age	1.034 (0.996–1.074)	0.078		
Sex	2.208 (0.890–5.475)	0.087		
Hypertension	0.686 (0.278–1.693)	0.413		
History of CAD	2.425 (0.11–55.178)	0.419		
History of COPD	0.529 (0.117–2.398)	0.409		
Concomitant AF	1.886 (0.767–4.636)	0.167		
AADs	1.736 (0.718–4.198)	0.221		
*Echocardiographic variables*				
LVEDV	0.996 (0.981–1.012)	0.640		
LVEF	0.982 (0.946–1.019)	0.339		
LAVi	0.995 (0.982–1.007)	0.395		
LASr	0.890 (0.828–0.957)	0.002	0.919 (0.852–0.992)	0.03
TAPSE	0.918 (0.825–1.023)	0.122		
RAVi	1.020 (0.994–1.046)	0.128		
RASr	0.878 (0.812–0.950)	0.001	0.904 (0.831–0.984)	0.02
At least moderate MR	0.460 (0.151–1.405)	0.173		
At least moderate TR	0.350 (0.109–1.121)	0.077		

Abbreviations: AADs, antiarrhythmic drugs; AF, atrial fibrillation; CAD, coronary artery disease; COPD, chronic obstructive pulmonary disease; LASr, left atrial strain reservoir; LAVi, left atrial volume index; LVEDV, left ventricular end diastolic volume; LVEF, left ventricular ejection fraction; MR, mitral regurgitation; RAVi, right atrium volume index; RASr, right atrial strain reservoir; RV, right ventricle; TAPSE, tricuspid annulus planar systolic excursion; TR, tricuspid regurgitation.

**Table 3:** Comparison between groups in sinus rhythm vs. AFL during the echocardiographic evaluation.

*Variables*	SR During Echocardiography 36 pts	AFL During Echocardiography 26 pts	p-Value (Sig.2-Tailed)
LVEDV (mL)	95.7 ± 28.5	103.6 ± 34.2	0.369
LVEF (%)	54 ± 13.5	45.7 ± 11.1	**0.005**
LAVi (mL/m^2^)	37.1 ± 11.2	42.7 ± 17.5	**<0.001**
LASr (%)	20.4 ± 9.3	17.8 ± 7.5	0.189
RV mid-diameter (mm)	29.1 ± 6.7	30.7 ± 5.5	0.149
TAPSE (mm)	18.9 ± 3.8	17.4 ± 4.3	0.277
RAVi (mL/m^2^)	22.9 (13.2–33.1)	31.2 (24.7–45.4)	**0.015**
RASr (%)	18.5 ± 7.9	16.5 ± 5.4	0.159
AA recurrence	11 (31%)	10 (38%)	0.516

*Abbreviations:* AAs, atrial arrhythmias; LVEDV, left ventricular end diastolic volume; LVEF, left ventricular ejection fraction; LA, left atrium; LAVi, left atrial volume index; LASr, left atrial strain reservoir; RV, right ventricle; TAPSE, tricuspid annulus planar systolic excursion; RA, right atrium; RAVi, right atrial volume index; RASr, right atrial strain reservoir; SR, sinus rhythm.

**Table 4:** Comparison between patients who received isolated CTIA and those who underwent concomitant AF ablation.

	Isolated CTIA 35 pts	CTIA + AF Ablation 27 pts	*p*-Value (Sig.2-Tailed)
*Clinical* *characteristics*			
Female sex (n, %)	12 (34.3%)	6 (22.2%)	0.299
Age (years)	62.9 ± 14.7	67.2 ± 10.7	0.356
BMI (kg/m^2^)	26.7 ± 3.9	27.3 ± 5.2	0.926
Hypertension (n, %)	18 (51.4%)	16 (59.2%)	0.539
Diabetes (n, %)	6 (17.1%)	3 (11.1%)	0.504
CAD (n, %)	4 (11.4%)	2 (7.4%)	0.595
COPD (n, %)	3 (8.5%)	3 (11.1%)	0.737
Beta-blockers (n, %)	27 (77.1%)	22 (81.4%)	0.468
AADs (n, %)	8 (22.9%)	14 (51.8%)	0.013
*Echocardiography*			
LVEDV (mL)	99.1 ± 33.2	99.5 ± 28.8	0.747
LVEF (%)	50.2 ± 13.9	50.8 ± 12	0.959
LAVi (mL/m^2^)	41.8 ± 13.9	40.2 ± 18.5	0.332
LASr (%)	18.9 ± 6.7	18.5 ± 10.7	0.444
TAPSE (mm)	18.1 ± 4.3	18.4 ± 3.8	0.653
RAVi (mL/m^2^)	33.2 (25–40)	32.1 (21.4–42)	0.551
RASr (%)	17.2 ± 6.7	18 ± 7.3	0.790
At least moderate MR	5 (14.3%)	2 (27.4%)	0.396
At least moderate TR	4 (11.4%)	2 (27.4%)	0.595

Abbreviations: AADs, antiarrhythmic drugs; AF, atrial fibrillation; CAD, coronary artery disease; COPD, chronic obstructive pulmonary disease; CTIA, cavotricuspid isthmus ablation; LASr, left atrial strain reservoir; LAVi, left atrial volume index; LVEDV, left ventricular end diastolic volume; LVEF, left ventricular ejection fraction; MR, mitral regurgitation; RAVi, right atrium volume index; RASr, right atrial strain reservoir; TAPSE, tricuspid annulus planar systolic excursion; TR, tricuspid regurgitation.

**Table 5:** Comparison between patients who experienced AFL and those who experienced AF at follow-up.

	AFL at Fup 11 pts	AF at Fup 10 pts	*p*-Value (Sig.2-Tailed)
*Clinical* *characteristics*			
Female sex (n, %)	5 (45.5%)	5 (50%)	0.835
Age (years)	67.8 ± 12.8	66.9 ± 11	0.863
BMI (kg/m^2^)	26.7 ± 3.4	28.3 ± 5.8	0.705
Hypertension (n, %)	5 (45.5%)	6 (60%)	0.781
Diabetes (n, %)	0 (0%)	4 (40%)	0.020
CAD (n, %)	0 (0%)	0 (0%)	0.065
COPD (n, %)	1 (9%)	1 (10%)	0.977
Concomitant AF ablation (n, %)	7 (63.6%)	6 (60%)	0.835
Beta-blockers (n, %)	8 (78%)	9 (80.9%)	0.314
AADs (n, %)	6 (26.8%)	5 (52.4%)	0.055
*Echocardiography*			
LVEDV (mL)	97.1 ± 21.3	94.2 ± 30	0.497
LVEF (%)	51.3 ± 12.3	47.9 ± 11.3	0.512
LAVi (mL/m^2^)	22.6 (12–35)	28 (13.2–41)	0.512
LASr (%)	13.2 ± 5.9	12.6 ± 4.5	0.973
TAPSE (mm)	17.8 ± 5.3	17.3 ± 3.4	0.863
RAVi (mL/m^2^)	29.25 (11.9–41.6)	28 (14.2–39.5)	0.809
RASr (%)	12.5 ± 4.8	13.2 ± 6	0.863

Abbreviations: AADs, antiarrhythmic drugs; AF, atrial fibrillation; AFL, atrial flutter; CAD, coronary artery disease; COPD, chronic obstructive pulmonary disease; LASr, left atrial strain reservoir; LAVi, left atrial volume index; LVEDV, left ventricular end diastolic volume; LVEF, left ventricular ejection fraction; RAVi, right atrium volume index; RASr, right atrial strain reservoir; TAPSE, tricuspid annulus planar systolic excursion.

## Data Availability

Data are available under request.

## Supplementary Materials

The following supporting information can be downloaded at https://www.mdpi.com/article/10.3390/jcm14155247/s1. Figure S1: Study flow-chart; Table S1. Population characteristics stratified by RASr values below or above ROC curve-derived cut-off value for AA recurrence; Table S2. Population characteristics stratified by LASr values below or above ROC curve-derived cut-off value for AA recurrence.
