# Supplementary material for: Atrial Strain Analysis Predicts Atrial Arrhythmia Recurrence Following Cavotricuspid Isthmus Ablation of Typical Atrial Flutter
Source: J Clin Med. 2025 Jul 24;14(15):5247. doi: 10.3390/jcm14155247 (PMC12348016; doi:10.3390/jcm14155247)
Supplement: Supplementary file 1 [file jcm-14-05247-s001.zip › jcm-3689633-supplementary.pdf]

Figure S1: Study flow-chart.

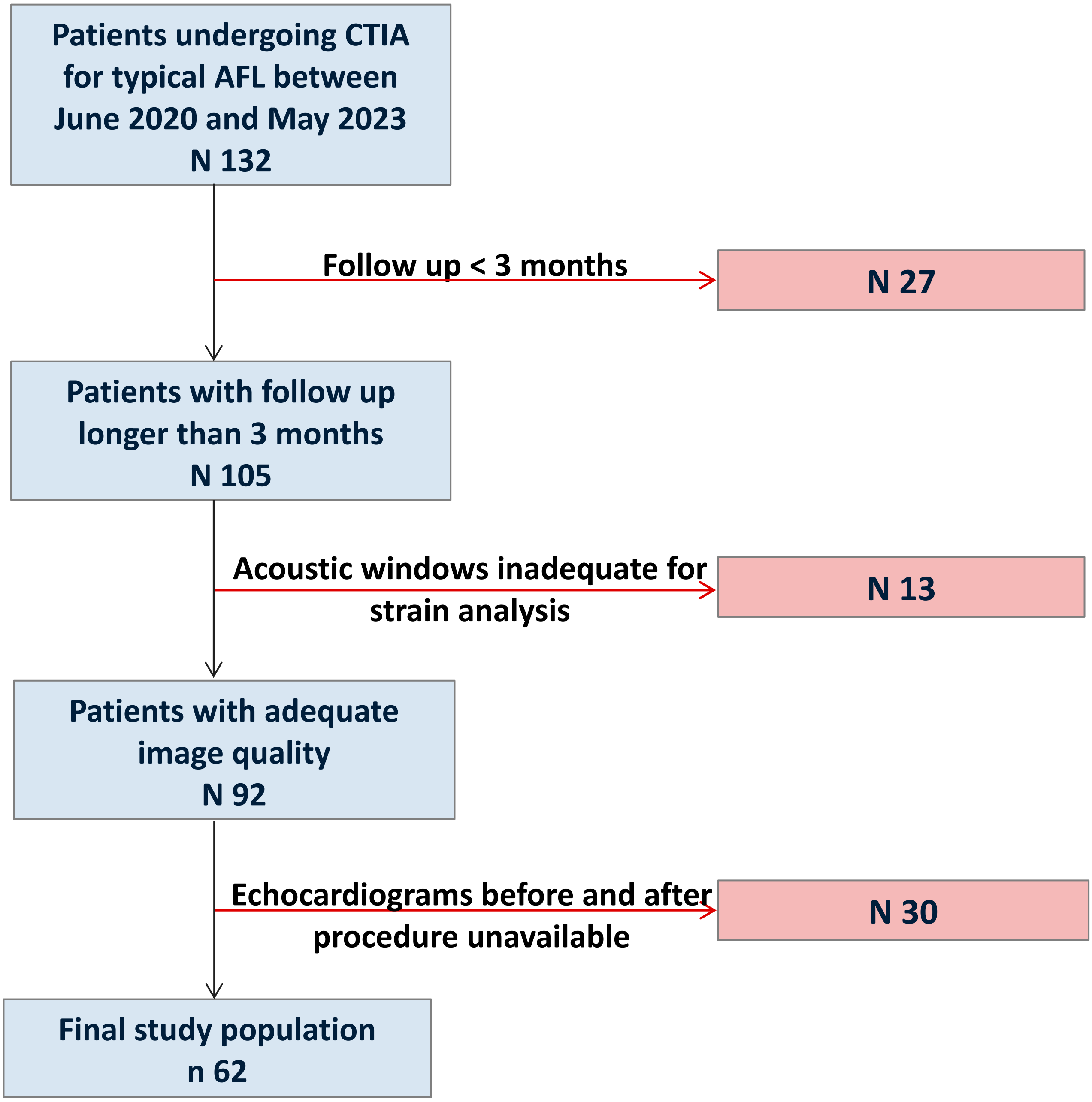

Abbreviations: CTIA: cavo-tricuspid isthmus ablation; AFL: atrial flutter.

Table S1. Population characteristics stratified by RASr values below or above ROC curve-derived cut-off value for AA recurrence.

	Overall population (n=62)	RASr < 16.8% (n=25)	RASr > 16.8% (n=37)	p-value (Sig.2-tailed)
Clinical characteristics				
Female sex (n,%)	18 (29%)	10 (40%)	8 (21.6%)	0.118
Age (years)	64.8 ± 13.2	67 ± 11.7	63.2 ± 14	0.265
BMI (kg/m2)	26.9 ± 4.5	27.7 ± 4.9	26.4 ± 4.1	0.260
Hypertension (n,%)	34 (58.4%)	15 (60%)	19 (51.5%)	0.502
Diabetes mellitus (n,%)	9 (14.5%)	7 (28%)	2 (5.4%)	**0.013**
Current smoker (n,%)	9 (14.5%)	4 (16%)	5 (13.5%)	0.887
CAD (n,%)	6 (9.7%)	2 (8%)	4 (10.8%)	0.713
COPD (n,%)	6 (9.7%)	2 (8%)	4 (10.8%)	0.713
Concomitant AF ablation (n,%)	27 (43.5%)	12 (48%)	15 (40.5%)	0.561
Beta-blockers (n,%)	49 (79%)	19 (76%)	30 (81.1%)	0.854
AAD (n,%)	22 (35.5%)	9 (40.9%)	13 (35.1%)	0.851
Creatinine (mg/dl)	1.1 ± 0.4	1.1 ± 0.5	1.1 ± 0.3	0.879
ECG features				
HR (bpm)	93.5 ± 33.2	93.6 ± 36.5	93.4 ± 36.5	0.986
QRS (ms)	119.2 ± 31	122.4 ± 24.4	117 ± 34.9	0.510
Echocardiography				
LVEDV (ml)	99.2 ± 31.1	91 ± 28.4	104.6 ± 32.1	0.104
LVEF (%)	50.5 ± 13.1	49.8 ± 12.4	50.9 ± 13.6	0.766
LAVi (ml/m2)	40.1 ± 15.9	43.4 ± 18	39.7 ± 13.6	0.063
TAPSE (mm)	18.2 ± 4.1	17.4 ± 4.2	18.9 ± 3.5	0.055
PASP (mmHg)	33.2 ± 8.5	33.3 ± 8	31.5 ± 9	0.497
RVFAC (%)	32.3 ± 11.1	30.7 ± 11.6	33.2 ± 10.8	0.415
RV mid diameter (mm)	29.8 ± 6.2	31.5 ± 6.2	29 ± 6.1	0.224
RAVi (ml/m2)	30.2 (21.4-41.5)	34.1 (22-40.5)	32 (18-45.5)	0.630

At least moderate MR (n,%)	7 (11.3%)	4 (16%)	3 (8.1%)	0.335
At least moderate TR (n,%)	6 (9.7%)	4 (16%)	2 (5.4%)	0.166

Abbreviations: AA: atrial arrhythmias; AAD: antiarrhythmic drugs; AF: atrial fibrillation; CAD: coronary artery disease; COPD: chronic obstructive pulmonary disease; LASr: left atrial strain reservoir; LAVi: left atrial volume indexed; LVEDV: left ventricular end diastolic volume; LVEF: left ventricular ejection fraction; MR: mitral regurgitation; PASP: pulmonary artery systolic pressure; RAVi: right atrium volume indexed; RASr: right atrial strain reservoir; ROC: receiver operator characheristics; RV: right ventricle; TAPSE: tricuspid annulus planar systolic excursion; TR: tricuspid regurgitation.

Table S2. Population characteristics stratified by LASr values below or above ROC curve-derived cut-off value for AA recurrence.

	Overall population (n=62)	LASr < 17.7% (n=28)	LASr > 17.7% (n=34)	p-value (Sig.2-tailed)
Clinical characteristics				
Female sex (n,%)	18 (29%)	11 (39.2%)	7 (20.5%)	0.107
Age (years)	64.8 ± 13.2	65.7 ± 13.7	64 ± 12.8	0.621
BMI (kg/m2)	26.9 ± 4.5	27.7 ± 5	26.3 ± 3.9	0.202
Hypertension (n,%)	34 (58.4%)	14 (50%)	20 (58.8%)	0.487
Diabetes mellitus (n,%)	9 (14.5%)	5 (17.8%)	4 (11.8%)	0.498
Current smoker (n,%)	9 (14.5%)	5 (17.8%)	4 (11.8%)	0.498
CAD (n,%)	6 (9.7%)	3 (10.7%)	3 (8.8%)	0.802
COPD (n,%)	6 (9.7%)	1 (3.6%)	5 (14.7%)	0.140
Concomitant AF ablation (n,%)	27 (43.5%)	15 (53.4%)	12 (35.3%)	0.149
Beta-blockers (n,%)	49 (79%)	21 (75%)	28 (82.3%)	0.655
AAD (n,%)	22 (35.5%)	13 (46.4%)	9 (26.5%)	0.056
Creatinine (mg/dl)	1.1 ± 0.4	1.08 ± 0.4	1.14 ± 0.4	0.549
ECG features				
HR (bpm)	93.5 ± 33.2	94.1 ± 35.3	93 ± 31.9	0.898
QRS (ms)	119.2 ± 31	118 ± 28.1	120.2 ± 33.6	0.788
Echocardiography				
LVEDV (ml)	99.2 ± 31.1	103.2 ± 33	96.4 ± 29.9	0.417
LVEF (%)	50.5 ± 13.1	48.5 ± 11.7	52 ± 14.1	0.296
LAVi (ml/m2)	40.1 ± 15.9	45.9 ± 18.6	37.3 ± 12.5	**0.036**
TAPSE (mm)	18.2 ± 4.1	18 ± 3.9	18.4 ± 4.3	0.689
PASP (mmHg)	33.2 ± 8.5	34.4 ± 9.3	30.6 ± 7.6	0.149
RVFAC (%)	32.3 ± 11.1	33.8 ± 13	31.1 ± 9.6	0.367
RV mid diameter (mm)	29.8 ± 6.2	29.2 ± 5.5	30.3 ± 6.7	0.496
RAVi (ml/m2)	30.2 (21.4-41.5)	34.2 (13.5-50)	31.7 (12-43.5)	0.550

At least moderate MR (n,%)	7 (11.3%)	4 (14.3%)	3 (8.8%)	0.499
At least moderate TR (n,%)	6 (9.7%)	2 (7.1%)	4 (11.8%)	0.079

Abbreviations: AA: atrial arrhythmias; AAD: antiarrhythmic drugs; AF: atrial fibrillation; CAD: coronary artery disease; COPD: chronic obstructive pulmonary disease; LASr: left atrial strain reservoir; LAVi: left atrial volume indexed; LVEDV: left ventricular end diastolic volume; LVEF: left ventricular ejection fraction; MR: mitral regurgitation; PASP: pulmonary artery systolic pressure; RAVi: right atrium volume indexed; RASr: right atrial strain reservoir; ROC: receiver operator characheristics; RV: right ventricle; TAPSE: tricuspid annulus planar systolic excursion; TR: tricuspid regurgitation.
